# Effect of Cadmium and Copper Exposure on Growth, Physio-Chemicals and Medicinal Properties of *Cajanus cajan* L. (Pigeon Pea)

**DOI:** 10.3390/metabo11110769

**Published:** 2021-11-10

**Authors:** Khizar Hayat, Asif Khan, Farkhanda Bibi, Salahuddin, Waheed Murad, Yujie Fu, Gaber El-Saber Batiha, Mohammed Alqarni, Ajmal Khan, Ahmed Al-Harrasi

**Affiliations:** 1 Key Laboratory of Plant Ecology, Northeast Forestry University, Harbin 150040, China; khizarhayatnefu637@gmail.com; 2 Department of Botany, Abdul Wali Khan University Mardan, Mardana 23200, Pakistan; asif.awkum.edu@gmail.com (A.K.); waheedmurad@awkum.edu.pk (W.M.); 3 Laboratory of Phytochemistry, Department of Botany, University of São Paulo, São Paulo 05508-090, Brazil; 4 CAS Key Laboratory of Tropical Plant Resources and Sustainable Use, Xishuangbanna Tropical Botanical Garden, Chinese Academy of Sciences, Menglun 666303, China; farkhanda@xtbg.ac.cn; 5 Agriculture Research Station, Harichand, Charsadda 24520, Pakistan; salaaup@gmail.com; 6 Department of Pharmacology and Therapeutics, Faculty of Veterinary Medicine, Damanhour University, Damanhour 22511, Egypt; gaberbatiha@gmail.com; 7 Department of Pharmaceutical Chemistry, College of Pharmacy, Taif University, P.O. Box 11099, Taif 21944, Saudi Arabia; m.aalqarni@tu.edu.sa; 8 Natural and Medical Sciences Research Center, University of Nizwa, Birkat Al Mauz, P.O. Box 33, Nizwa 616, Oman

**Keywords:** metals cumulative stress, oxidative damage, antioxidant enzymes, medicinal properties, pigeon pea

## Abstract

Soil contamination with heavy metals is an emerging concern in the modern era, affecting all forms of life. Pigeon pea is a multi-use shrub with medicinal and nutritional values. On the basis of a randomized complete design, we investigated in the current project the combined cadmium (Cd) and copper (Cu) effect on plant growth and physio-chemical/medicinal properties of pigeon pea. Three-week-old seedlings were grown in combined Cd and Cu amended soil with increasing metal concentrations (control, 20 + 30 mg/kg, 40 + 60 mg/kg, and 60 + 90 mg/kg) for three months. At high-dose metal cumulative stress (60 + 90 mg/kg), plant shoot and root growth in terms of plant height as well as fresh and dry weight were significantly inhibited in association with decreased photosynthetic attributes (chlorophyll a and b contents, net photosynthesis, transpiration rate, stomatal conductance, intercellular CO_2_ concentrations) and diminished nutrient contents. Cd and Cu at high amounts inflicted oxidative stresses as assessed in elevated lipid peroxidation (MDA), hydrogen peroxide (H_2_O_2_), and electrolyte leakage contents. Antioxidant enzyme activities, namely, those of superoxide dismutase (SOD), catalase (CAT), peroxidase (POD), and glutathione peroxidase (GPX), were enhanced, along with proline content with increasing metal quantity. Phenolics and flavonoids exhibited a diverse response regarding metal concentration, and their biosynthesis was significantly suppressed at high Cd and Cu cumulative stress. The reduction in secondary metabolites may account for declined medicinal properties of pigeon pea as appraised in reduced antibacterial, 2, 2-diphenyl-1-picrylhydrazyl (DPPH), and ferric-reducing antioxidant potential (FRAP) activities. Our results clearly demonstrate that the exposure of pigeon pea to Cd- and Cu-contaminated soil might affect consumers due to the presence of metals and the negligible efficacy of the herbal products.

## 1. Introduction

Soil contamination with heavy metals is a widespread environmental issue, originating from industrial growth, urbanization, agriculture practices, mining activities, and municipal waste [1,2]. These pollutants adversely affect the surrounding environment, reduce agricultural productivity, and cause severe health hazards to living organisms [3,4]. Among all heavy metals, cadmium (Cd) and copper (Cu) are of main concern, owing to their higher mobility, non-degradability, and toxicity, which affects animals and plants [5]. Cadmium (Cd) does not have any physiological role in plant metabolism and is very toxic, even at low concentrations. Its contamination sources include lithogenic, pedogenic, and anthropogenic sources that release approximately 1–70, 11,000, and 16,000 metric tons per annum of cadmium into the biosphere, respectively [6,7]. Cd^2+^ excessive accumulation in plants might cause severe phytotoxicity and numerous physiological, morphological, and biochemical toxic effects on plant attributes such as pigment destruction, photosynthetic and respirational process inhiations, lessening nutrient uptake, overproduction of reactive oxygen species (ROS), enzyme and gene suppression, growth inhibition, and even plant death [8,9,10,11,12,13].

Copper (Cu) is an essential micronutrient for plants since it contributes to different physiological processes of plants, including mitochondrial respiration, photosynthetic electron transport, cell wall metabolism, DNA transcription, protein trafficking, hormone signaling, and protein regulation [14,15,16]. However, in excessive amounts, it is toxic for plants because of its redox properties [17]. However, it’s in excessive amount inhibits plant growth, affects photosynthetic and respiratory processes, decreases nutrients uptake, targets the membrane transport system, and produces ROS in undue quantities [18,19,20,21]. Its contaminations sources include industrial waste, copper mining, anti-fouling paints, farming practices, copper-based pesticides, and copper marine drainage [22,23].

Plant exposure to metals stress generates reactive oxygen species (ROS) in an excessive amount that inflicts oxidative stresses [24]. Oxidative stresses disturb cellular redox balance and damage delicate cellular entities such as DNA molecules, proteins, and membranes [25,26]. To minimize metals induce damages, plants have evolved different strategies including metal exclusion; compartmentalization; chelation; and a wide spectrum of ROS-scavenging mechanisms, including antioxidant enzymes such as superoxide dismutase (SOD), catalase (CAT), peroxidase (POX), glutathione reductase (GR), ascorbate peroxidase (APX), as well as non-enzymatic antioxidants including phenolics, flavonoids, proline, ascorbate (AsA), glutathione (GSH), along with an array of stress mitigation molecules [27,28,29,30,31]. Plants’ secondary metabolites not only perform their role in plant adaptation to the specific environment, but also are an important source for pharmaceutical-related drugs [30]. Thus, any environmental contaminant that causes fluctuations in these photochemicals might affect the medicinal properties of its derivatives plants [32,33].

Pigeon pea (*Cajanus cajan* L.), a member of Fabaceae, is grown mostly in the tropical region of the world. The plant body is erect, branched with oblanceolate leaves and having yellow flowers, with versatile properties including use as a nutritional supplement as well as for medicinal purposes. Its seeds serve as an affluent protein source, pods as a vegetable, and leaves and husks as silage. Moreover, its extracts display strong anti-bacterial, anti-viral, anti-diabetic, anti-malarial, anti-fungal, anti-inflammatory, anti-cancer, and antioxidant action due to the presence of various classes of phytochemicals such as phenolics, saponins, alkaloids, flavonoids, and stilbenes [34,35,36,37,38,39,40]. To the best of our knowledge, previously, no work has been carried out on the interactive effect of Cd and Cu on *Cajanus cajan*; hence, the present experiment was conducted in order to explore the combined Cd and Cu effect on plant physiological response, oxidative stresses, and growth in association with its medicinal properties (antibacterial and anti-oxidant actions).

## 2. Results

### 2.1. Plant Growth Attritubutes

The results of one-way ANOVA showed significant effect of metals’ combined treatment effect on pigeon pea shoot length, root length, and biomass, which are given in Table 1. Plant growth attributes were decreased with increasing Cd and Cu contents in soil. As compared to the control, maximum reductions in the shoot length (19.69%), fresh weight (34.28%), and dry weight (37.18%) were observed at higher dose of Cd and Cu combined stress, i.e., Cd 60 + Cu 90 mg/kg. Similarly, the root attributes, including root length, fresh mass, and dry mass were decreased by 42.52%, 29.36% and 30.26% respectively at higher Cd and Cu combined stress (60 + 90 mg/kg) as compared to control.

### 2.2. Chlorophyll Content and Photosynthetic Parameters

**Figure 1:**
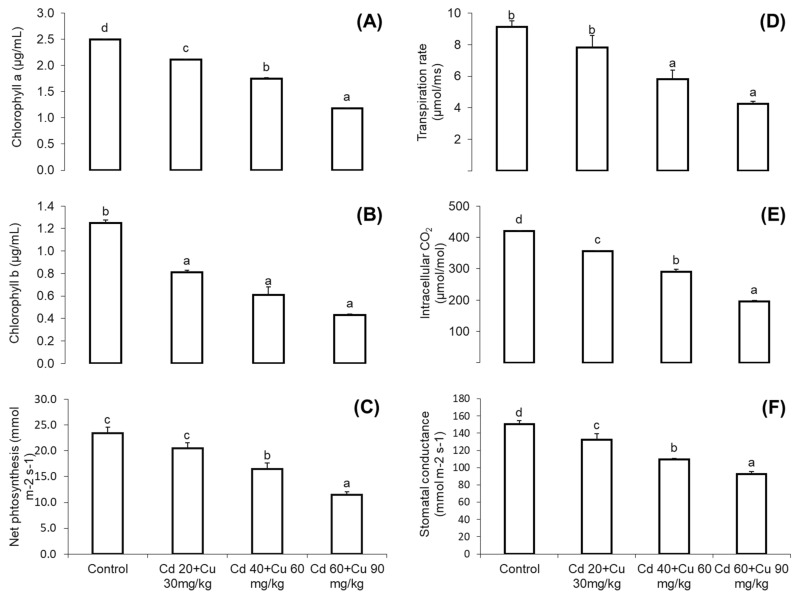
The effect of different Cd and Cu concentrations (0, Cd 20 + Cu 30 mg/kg, Cd 40 + Cu 60 mg/kg, and Cd 60 + Cu 90 mg/kg) on the chlorophyll a (**A**), chlorophyll b (**B**), net-photosynthesis (**C**), transpiration rate (**D**), intercellular CO_2_ (**E**), and stomatal conductance (**F**) on the leaves of the pigeon pea. Different letters represent significant differences between the treatments at *p* ≤ 0.05.

It was taken into consideration that metals combined stress at high concentration (Cd 60 + Cu 90 mg/kg) adversely affect the photosynthetic parameters, i.e., chlorophyll a and b, net photosynthetic rate, transpiration rate, stomatal conductance, and internal CO_2_ concentrations (Figure 1). There was a significant difference among different treatments with respect to the control, and the chlorophyll contents significantly declined with the increasing concentration of metals in the soil. Maximum reduction of 52.61% in the chlorophyll a, 42.27% in chlorophyll b, and 51.17% in the net photosynthesis were measured at a high dose in the metal-treated plants with respect to the control (Figure 1A–C). Similarly, Cd and Cu substantially reduced the transpiration rate, internal CO_2_ concentration, and stomatal conductance. A maximum reduction of 23.5% was noted in the transpiration rate, followed by 54.9% in the internal CO_2_ concentration and 38.53% in the stomatal conductance at Cd 60 + Cu 90 mg/kg amended soil plants as compared with the control (Figure 1D–F).

### 2.3. Flavonoid, Phenolic, and Proline Contents

Cd and Cu combined effect on secondary metabolites (phenolic and flavonoids) in the leaves of pigeon pea were monitored (Table 2). Secondary metabolites displayed diverse responses with regard to metals concentrations in soil. The plants grown on low Cd and Cu amended soil (20 + 30 mg/kg), flavonoid and phenolic production was significantly increased by 28.42 and 23.45%, respectively, as compared to the control. On the other hand, the increasment of metals in soil significantly reduced secondary metabolites productions. Maximum reduction of 46.18% in flavonoids and 41.52% in phenolics was measured in plants exposed to combined Cd and Cu stress (60 + 90 mg/kg). Cd and Cu impact on proline contents are given in Table 2. Proline contents were significantly increased with increasing Cd and Cu stress as compared to control. Where, maximum increase of 342% was observed in plants exposed to combined Cd and Cu stress (60 + 90 mg/kg).

### 2.4. Medicinal Properties (Anti-Bacterial, DPPH, and FRAP Activity)

**Figure 2:**
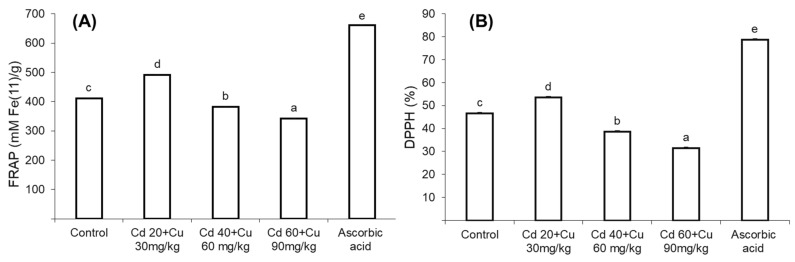
The impact of different Cd and Cu stress concentrations (0, Cd 20 + Cu 30 mg/kg, Cd 40 + Cu 60 mg/kg, and Cd 60 + Cu 90 mg/kg) on the shoot’s FRAP (**A**) and DPPH (**B**) contents of pigeon pea plants. Bars represent means from the three independent replicates (±, *n* = 3); different letters indicate significant differences between the treatments at *p* < 0.05.

Collective Cd and Cu effect on *Cajanus cajan* L medicinal properties in terms of its anti-bacterial activity, 2,2-diphenyl-1-picrylhydrazyl (DPPH) level, and ferric reducing antioxidant power assay (FRAP) is shown in Figure 2.

In general, the plants grown on low Cd and Cu amended soil (20 + 30 mg/kg) extracts exhibited slightly higher anti-bacterial inhibition zone (12.93, 11.22, and 13.75 mm) against *S. aureus, E. coli*, and *S. thyphi* strains, respectively, compared to control plants’ inhibition zone (11.45 mm, 10.52 mm, and 12.43 mm, respectively) (Table 3). On the other hand, addition of Cd and Cu at higher concentrations in the soil significantly reduced the antibacterial activity of *Cajanus cajan* L. Maximum reduced bacterial inhibition zone was observed in 60 + 90 mg/kg plant extract (9.65, 8.44 and 8.21 mm) as compared to control plants. Likewise, the plants grown in low Cd and Cu amended soil (20 + 30 mg/kg) extract showed enhanced 2, 2-diphenyl-1-picrylhydrazyl (DPPH) and ferric reducing antioxidant power (FRAP) (Figure 2A,B). However, increasing metal content in soil gradually reduced plant extracts’ DPPH and FRAP antioxidant power as compared to control.

### 2.5. Oxidative Stresses

**Figure 3:**
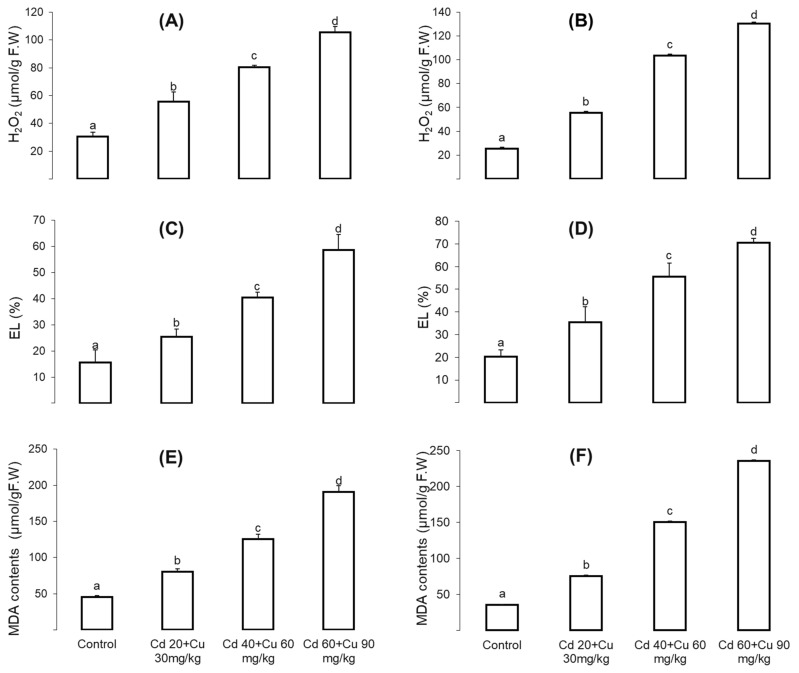
The effect of different Cd and Cu treatments (0, Cd 20 + Cu 30 mg/kg, Cd 40 + Cu 60 mg/kg, and Cd 60 + Cu 90 mg/kg) on the shoot (**A**,**C**,**E**) and root (**B**,**D**,**F**) H_2_O_2_, EL, and MDA contents of pigeon pea plants. Bars represent means from the three independent replicates (±, *n* = 3); different letters indicate significant differences between the treatments at *p* < 0.05.

Combined Cd and Cu impact on oxidative stress indicators, i.e., malondialdehyde (MDA), hydrogen peroxide (H_2_O_2_), and electrolyte leakage (EL) contents in the *Cajanus cajan* shoot and roots were monitored (Figure 3). The results revealed that these oxidative stress indicators were significantly increased with increasing metal contents in the soil. At the highest Cd and Cu combined stress (60 + 90 mg/kg), the H_2_O_2_, EL, and MDA contents in the shoot were increased by 246.76%, 278.29%, and 319.18%, respectively (Figure 3A,C,E), while in the root, malondialdehyde (MDA), hydrogen peroxide (H_2_O_2_), and electrolyte leakage (EL) were increased by 412%, 323% and 247.38% respectively as compared to the control (Figure 3B,D,F).

### 2.6. Antioxidant Enzyme Response

**Figure 4:**
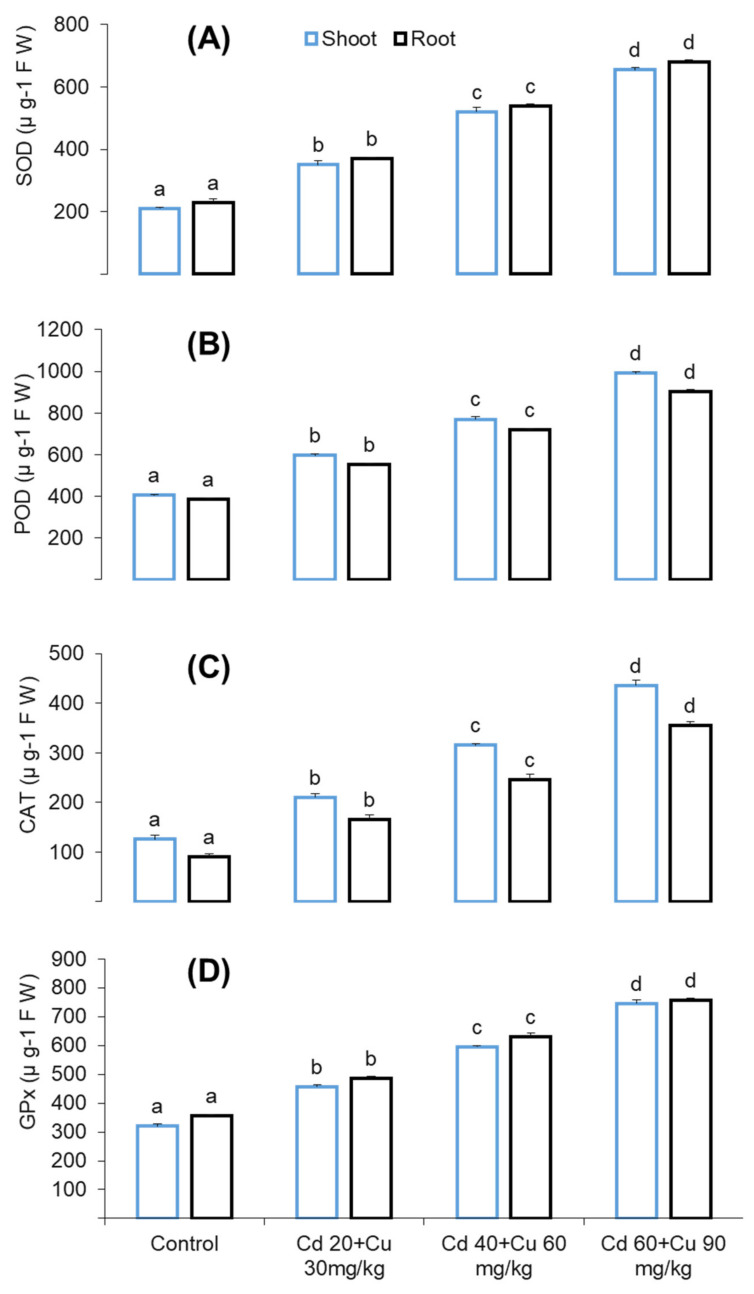
The effect of different cadmium and copper concentrations (0, Cd 20 + Cu 30 mg/kg, Cd 40 + Cu 60 mg/kg, and Cd 60 + Cu 90 mg/kg) on shoots and roots’ antioxidant enzymes, namely, SOD (**A**), POD (**B**), CAT (**C**), and GPX (**D**) contents in pigeon pea plant. Bars represent means from the three independent replicates (±, *n* = 3); different letters indicate significant differences between the treatments at *p* < 0.05.

Antioxidant enzymes such as superoxide dismutase (SOD), peroxidase (POD), catalase (CAT), and glutathione peroxidase (GPX) response were checked under different Cd and Cu combined stresses (Figure 4). As compared to the control, the activities of these enzymes were significantly enhanced with respect to the metal increasing quantity in the soil. At the highest Cd and Cu cumulative stress (60 + 90 mg/kg) SOD, POD, CAT, and GPX activities in leaves were increased by 211.74%, 144.35%, 247.11%, and 132.64%, respectively, while in the roots, this increase was 196.16%, 132.98%, 100.16%, and 124.85% as compared to control (Figure 4 A–D).

### 2.7. Metal (Cd and Cu) Accumulation

**Figure 5:**
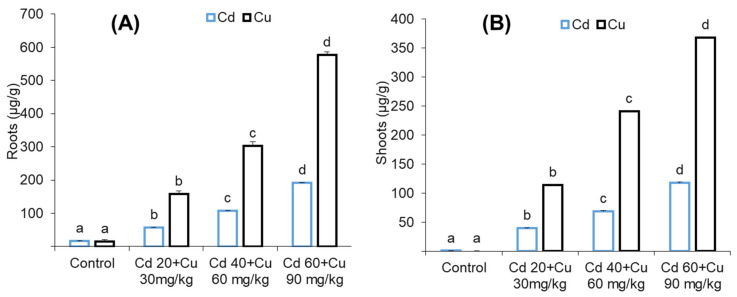
The effect of different cadmium and copper concentrations (0, Cd 20 + Cu 30 mg/kg, Cd 40 + Cu 60 mg/kg, and Cd 60 + Cu 90 mg/kg) on shoot (**A**) and root (**B**) metal accumulation of cadmium and copper in pigeon pea plant. Bars represent means from the three independent replicates (±, *n* = 3); different letters indicate significant differences between the treatments at *p* < 0.05.

The accumulation of cadmium (Cd) and copper (Cu) in pigeon pea root and leaves tissues are given in (Figure 5). It was observed that Cd and Cu mainly accumulated in the roots as compared to leaves in all Cd and Cu combined treated plants. Maximum Cd accumulated i.e., (191.45 μg/g), Cu (576.52 μg/g) were detected in roots, and (117.48 μg/g), (367.55 μg/g) in the leaves were detected in plants exposed to highest Cd and Cu combined treatment (60 + 90 mg/kg).

### 2.8. Nutrient Uptake

**Figure 6:**
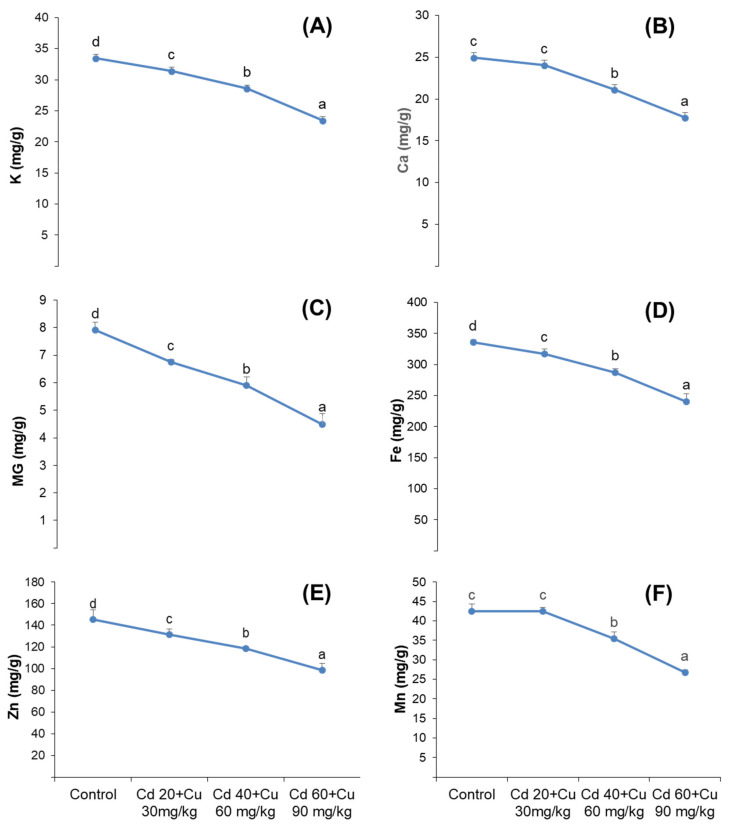
Effect of different cadmium and copper concentrations (0, Cd 20 + Cu 30 mg/kg, Cd 40 + Cu 60 mg/kg, and Cd 60 + Cu 90 mg/kg) on shoot macro-nutrient K^+^, Ca^+^, and Mg^2+^ (**A**–**C**) and macro-nutrient Fe^+^, Zn, and Mn (**D**–**F**) contents in pigeon pea plant. Bars represent means from the three independent replicates; different letters indicate significant differences between the treatments at *p* < 0.05.

Cadmium and copper combined effect on distribution of macro-nutrients (K^+^, Ca^+^, and Mg^2+^), and micro-nutrients (Fe^+^, Zn and Mn) in pigeon pea is shown in Figure 6. Cadmium (Cd) and Copper (Cu) cumulative stress significantly inhibited both macro and micro nutrients distributions among the plant’s organs, where maximum reductions of 29.98% in K^+^,28.76% in Ca^+^ and 43.23% in Mg^2+^ was detected at (60 + 90 mg/kg) Cd and Cu combined stress in the leaves of pigeon pea. Similarly, 28.30%, 32.28%, and 37.14% reduction in the micro-nutrients (Fe^+^, Zn, and Mn, respectively) were monitored in the leaves of pigeon pea exposed to (60 + 90 mg/kg) Cd and Cu stress.

## 3. Discussion

Metal toxicity in plants causes growth inhibition and biomass reduction [1,2]. Though, these declines are mainly associated with species type, metal nature, the contaminant’s quantities in the soil, and time exposure. Worldwide, agricultural land is progressively contaminated with Cd and Cu, which reduces crop yields and creates health concerns among the consumers [3,4,5,6]. Cu is essential for the plant’s growth and development in a very minute quantity between 15 and 20 μg/g, Whereas Cd has no known physiological role in the plants and is considered to be one of the most damaging threats to plants, even at very small quantity i.e.,0.7 µg/g [7,8]. Recently, it was taken into consideration that due to the rapid increases of industrialization, particularly in China, in last few years, the concentrations of Cd and Cu are noticeably increasing in the soil, causing severe damage in plants, even at very low concentrations [9,10,11,12]. In our previous studies, different physiological and growth attributes of *Cajanus cajan* L. and *Cicer arietinum* L. plants were monitored under different Cd-stressed environments [13,14]. Generally, plants have the capability to cope with a stressed environment in limited conditions; thus, the aim of this study was to check the medicinal, physiological, and growth parameters of *C. cajan* L. plant exposure to increasing Cd and Cu combined treatment. It was found that these metals significantly affected growth in terms of repressed stem and root length as well reduced biomasses (Table 1). These results were quite similar to our previous articles [15,16,17,18], wherein similar findings were also noticed accordingly. The possible cause of such reduction might be the association of Cd and Cu with the cell wall and middle lamellae, which enhances the pectin’s cross-linkage [19,20]. Furthermore, the reduced photosynthetic activities (Figure 1), decreased nutrient content (Figure 6), and excessive ROS production under Cd and Cu exposure might also contribute to growth inhibition. Our obtained results are consistent with the previous findings, where similar reduced growth parameters were observed under Cd and Cu exposure [21,22].

Photosynthesis is the life driving process often at risk to metal stresses. Chlorophyll plays an essential role in light absorption, transmission, and translation into chemical energy at the light phase of photosynthesis. Heavy metals, particularly Cd, have been reported in chlorophyll degradation by inhibiting its biosynthetic enzymes such as protochlorophyllide reductase and δ-aminolaevulinic acid dehydratase [23]. In our results, chlorophyll a and b contents, net photosynthetic rate, transpiration rate, stomatal conductance, and internal CO_2_ concentrations were inhibited severely, even at very low Cd and Cu concentrations. Our observation was consistent with that of previous findings, where similar inhibition in photosynthetic attributes have been reported in other plant species [24,25]. The reason might have been due to the binding nature of Cd and Cu that might substitute the Mg^2+^ molecule of chlorophyll, thus decreasing its light absorption capacity. Moreover, the presence of these metals at the photosynthetic apparatus and decrease of CO_2_ partial pressure in the stroma lead to the closure of stomata and reduce the transpiration rate, stomatal conductance, and internal carbon dioxide concentration [25].

Metal uptake and distribution in a plant depends on the species, the metal concentration in the growth medium, and the plant’s exposure to metal stress [26,27]. In our results (Figure 5), pigeon pea roots retained higher Cd and Cu content compared to leaves. Our results are in line with previous findings, wherein similar metal retention in the root and leaves has been reported [28,29,30]. This illustrated the slow translocation of metals among plant tissues, while the higher contents of Cd and Cu in the root might also have been due to the direct exposure in the soil, compartmentalization in vacuoles, and cross-linkage of cadmium and copper with the carboxyl group of the cell wall protein and their interaction with protein thiol groups [31,32].

Plants require mineral nutrients in an appropriate quantity for growth and other vital physiological and biochemical process. Increasing cadmium and copper concentrations in the soil gradually decreased secondary metabolite biosynthesis (phenolics and flavonoids) in pigeon pea (Table 2). These predictions were quite similar to previous findings of Printz et al. and Khan et al., who claimed that the presence of Cd and Cu in the growing medium at elevated quantity affects the absorption and transport of macro- and micronutrients [33,34]. These metals, particularly Cd, have been reported to decrease membrane permeability by altering its H^+^-ATPase activities [35]. Furthermore, the existence of cadmium and copper in a plant’s body compete with other minerals in apoplast and root vacuoles and thus decrease their transport among plant organs. This might be due to the suppression of genes that participate in phenolic and flavonoids production [36]. Secondary metabolites are of great importance on account of their medicinal properties such as antimicrobial, anti-inflammatory, and antioxidant activities [37]. The reduced contents of phenolic and flavonoids (Table 2) affected the medicinal properties of pigeon pea, as assessed in terms of its reduced anti-bacterial and antioxidant activity (Table 3, Figure 4). Similar reduced phenolic and flavonoid contents have been reported by Ibraham et al. and Okem et al., under Cd, Cu and Cd, and Al stress in Sambung Nyawa (*Gynura procumbens* Lour.) and *Drmia elata* (Jacq.) species, respectively [38,39].

Besides plant’s secondary metabolites, proline is also an important osmole, being responsible for stress mitigation. Similarly, the proline contents were considered mandatory in order to know its quantity in *C. cajan* plant under different Cd and Cu concentrations in the soil. In our results, the proline contents were found to be significantly stimulated, even at high concentrations (60 + 90 mg/kg) of Cd and Cu (Table 2); similar findings were also reported by [40,41,42]. This substantial increase of proline contents might have been due to slow protein oxidation and increased glutamate synthesis rate. Consistent with our results, the previous findings’ increased proline contents were observed under Cd and Cu combined stress [43]. In this vein, it is known that plants adapt several tactics to counteract and detoxify oxidative damage under metals stresses such as accumulation of non-enzymatic antioxidants such as ascorbate (AsA), glutathione (GSH), and proline. In a stressful environment, proline regulates cellular redox potential, maintains osmotic balance, scavenges free radicals, and sustains photo-system 11 in photosynthetic chain reaction [44,45].

Plants’ aerobic metabolism produces ROS as a by-product, which plays a vital role in homeostatic and cell signaling [46]. However, in metal stress environment this ROS production level exceed its normal rate which leads to oxidative damage. The consequences of oxidative damage results in membrane leakage, DNA damage, enzymes inhibition and photosynthesis suppression. In the present study, exposure of pigeon pea at increasing Cd and Cu combined stress significantly increased MDA contents, hydrogen peroxide (H_2_O_2_) and electrolyte leakage (EL) (Figure 3). Similar oxidative damage has been reported in other plant species under Cd and Cu combined stress, [47,48,49]. ROS are of several types such as superoxide (^•^O^−^_2_), hydrogen peroxide (H_2_O_2_), and hydroxyl radical (^•^OH). Among them all, superoxide (^•^O^−^_2_) is highly unstable and extremely reactive, originating from molecular oxygen (O_2_) reduction and acting as the precursor to other reactive oxygen species [50]. Likewise, hydrogen peroxide (H_2_O_2_) is formed from the same synthetic channel, and it is comparatively stable in comparison to other ROS molecules to a certain extent under normal cellular conditions. It will act as a dual molecule on its production rate, either as a signaling or oxidative stress inducer [51]. Plants activate antioxidant enzymes’ defense scheme in opposing metal induce oxidative stress, therefore contributing a leading role in plants’ physiological defense mechanism against ROS-induced oxidative damage [52,53]. Antioxidant enzymes are of several types, and each of them performs different functions, such as superoxide dismutase (SOD), which helps in the reduction of superoxide radicals (^•^O^−^_2_) into hydrogen peroxide (H_2_O_2_), which is further scavenged by ascorbate peroxidase (APX), catalase (CAT), and glutathione peroxidase (GPX) into a final H_2_O molecule through a series of oxidation reduction chain mechanisms [53].

## 4. Materials and Methods

### 4.1. Material and Growth Conditions

The pot experiment was conducted in the greenhouse at Northeast Forestry University Harbin China. Greenhouse growth conditions were maintained as follows: 28/21 °C temperature (day/night), a 14 h photoperiod per day, 65–75% relative humidity, and 410–570 m^−2^ s^−1^ average daily photosynthetic active radiation. Pigeon pea seeds were properly ordered from the Chinese Medicinal University. Healthy seeds were primarily sterilized for 30 s with 80% ethanol and transferred to a 5% sodium hypochlorite (NaOCl) solution for 15 min and swabbed three times with de-ionized water. After sterilization, seeds were sown in pots (9 cm in height, 13 cm in diameter) containing a soil mixture of vermiculite and peat (1:4, *w*/*w*) mixed with sand (3:1, *w*/*w*) for germination.

### 4.2. Soil Preparation and Experimental Designs

Soil was collected from the botanical garden (1–27 cm depth) at Northeast Forestry University Harbin, China. The fine powder soil was dried for 7 days and ground with the help of a pestle and mortar and passed through 2 mm sieve tubes. Soil basic characteristics were determined by following the work of Sparks et al. [54]. Its physio-chemical properties were as follows: clay (72.8%); silt (11.2%); soil (13.4%); pH (6.4); electrical conductivity (2.3 mS/cm); organic matter (14.52 g/kg); available phosphorus (64.63 mg/kg); available potassium (79.39 mg/kg); total nitrogen (75.62 mg/kg); total copper (13 mg/kg); and soil Cd (0.09 mg/kg).

All pots were filled equally with soil and organized in the following complete randomized block design (CRBD) with three replications. After 21 days of germination, uniform seedlings were transferred to each single pot carefully. In the case of the control, the water was provided on a daily basis in order to maintain soil moisture at 75–85%. However, in the case of treatment, soil was artificially spiked with an increasing combination of CdCl_2_ and CuSO_4_ solutions as follows: 0, 20 + 40, 40 + 60, 60 + 90 mg/kg.

### 4.3. Determination of Gaseous Exchange

Determination of gaseous exchange, such as net photosynthesis (Pn), transpiration rate (E), stomatal conductance (Gs), and intercellular CO_2_ concentration (Ci), was monitored at healthy, top, and fully expanded young leaves with the help of a portable gas exchange system (Li-Cor model 6200, Lincoln, Dearborn, MI, USA). The whole procedure was conducted on a clear day’s with average temperature (25–29 °C), relative air moisture (67–73%), from 10.30 to 12.30 a.m. and 2.30 to 4.30 p.m. Leaves were supplied illumination from the red-blue LED light source. Leaf chamber temperature was kept at room temperature with 410 ppm of CO_2_ concentrations and a photosynthetic photon flux density (PPFD) of 680 mol photon/m^2^s^1^ [55].

### 4.4. Chlorophyll a and b Determination

For chlorophyll determinations, 500 mg fresh leaves were ground in 80% acetone with the help of a pestle and mortar and homogenized at 1000 rpm for 1 min. The homogenate was gathered and filtered, and the filtrate was centrifuged at 2500× *g* at 4 °C for 10 min. Absorbance was taken at 663 and 645 nm through a UV spectrophotometer (Lab Digital China) for chlorophyll *a* and *b* against blank of 80% acetone. Total chlorophyll a and chlorophyll b contents were calculated by the Lichtentaler equations [56]

Chlorophyll a = [12.7(OD_663nm_) − 2.69(OD_645nm_)] × (V/W) (1)

Chlorophyll b = [21.7(OD_663nm_) −4.57(OD_645nm_)] × (V/W) (2)

### 4.5. Determination of Proline Contents

Proline contents were determined according to the method of Bates et al. [57]. Fresh leaves of 500 mg were ground, then homogenized in 10 mL of 10% sulfo-salicylic acid and centrifuged at 90,000× *g* for 1 min. A total of 2 mL of the supernatant was mixed with an equivalent volume of ninhydrin and acetic acid. The mixture was incubated at 100 °C for 60 min and kept in a separating funnel. Subsequently, 4 mL of toluene was added, and the mixture was vigorously shaken until a pink layer appeared. Absorbance was calculated at 520 nm with the help of a UV spectrophotometer. The proline concentrations were estimated with the help of the following equation and are expressed as µg^−1^ fresh mass.

Total proline contents = (Abs _extract_ − blank)/slope × Vol _extract_/Vol _aliquot_ × 1/FW (3)

### 4.6. Determination of Total Phenolics and Flavonoids

Fresh leaves (500 mg) were ground, and samples were extracted with 10 mL of 80% ethanol on an orbital shaker at 50 °C for 120 min. The blend was filtered (Whatman™ No.1, Maidstone, UK), and the filtrate was used to estimate total phenolics and flavonoids.

Total phenolic contents of leaf extract were determined by Folin–Ciocalteu reagent following the procedure of Singleton and Rossi [58]. Sample extract (1 mL) and 1 mL of Folin–Ciocalteu reagent were mixed in a 10 mL test tube, followed by the addition of 1 mL of saturated sodium carbonate (35%). After 3 min, the blend was diluted with 7 mL distilled water and incubated for 90 min in the dark at room temperature. The absorbance was measured at 725 nm by a UV spectrophotometer against a blank using gallic acid as a standard. The results were expressed in ‘mg’ of gallic acid equivalents (GAE) per gram of dry leaf extract.

Total flavonoid contents in the leaf extracts were estimated by the method of Zhishen et al. [59]. Leaf extracts of 1 mL and 0.3 mL (NaNO_3_) were mixed in an aluminum foil-covered test tube and allowed to stand for 5 min. We further added 10% AlCl_3_ (0.3 mL) into the test tube, followed by 1 mM NaOH (2 mL). At 510 nm, the absorbance was measured against blank by UV spectrophotometer using as a standard. The results are shown as mg/g of the Rutin dry sample.

### 4.7. DPPH Free Radical Scavenging Activity

DPPH (2, 2-diphenyl-1-picrylhydrazyl) free radical scavenging activity of plant samples was measured by Mensor et al. [60]. DPPH 0.1 mM solution was prepared in methanol, and its initial absorbance was checked at 518 nm. Samples extract of 40 µL was added to DPPH methanolic solution (3 mL) and kept in the dark for 30 min, and the difference in the absorbance was made at 518 nm by UV spectrophotometer. Ascorbic acid (synthetic antioxidant) was used as a positive control. The DPPH (%) activity was calculated by using the following equation,

AA% = 100 − (Absorbance of Sample-Absorbance of blank) × 100)/Absorbance of control(4)

### 4.8. FRAP (Ferric-Reducing Antioxidant Power) Assay

FRAP assay was measured following Benzie and Strain’s method [61]. Briefly, 300 mM sodium acetate buffer (pH 3.6), 10 mM TPTZ solution in 40 mM HCl, and 20 mM solution of FeCl_3_·6H_2_O were mixed at a ratio of 10:1:1 to prepare FRAP reagents. Different sample extracts (50, 100, 150, 200, 250 µg/mL) were added to FRAP reagents (3 mL). The reaction mixture was kept in a water tube for 30 min at 37 °C; an increase in absorbance was measured at 593 nm by UV spectrophotometer and compared with ascorbic acid (synthetic antioxidant). Different FeSO_4_ solutions formulated a calibration curve. Ferric reducing antioxidant power (FRAP) ability was calculated from the following equation and is expressed in µM Fe (II)/g dry mass:

FRAP Value = (Change in absorbance of Sample/Change in absorbance of blank) × Absorbance of standard (ascorbic acid)(5)

### 4.9. Anti-Bacterial Assay

The anti-bacterial assay of plant leaves crude extracts was performed by the disc diffusion method [62]. Three bacterial strains, including one Gram-positive (*Staphylococcus aureus*) and two Gram-negative (*Escherichia coli* and *Staphylococcus typhi*) were obtained and cultured on nutrient agar. The bacterial density was standardized with the help of McFarland 0.5 turbidity standard and wiped on Mueller–Hinton agar surface. A total of 3 mg of sample extract was dissolved in 10 mL methanol and loaded onto sterile Whatman ^No.1^ filter paper discs (6 mm) that were permeated onto inoculated agar. The discs were allowed for extract diffusion at 4 °C for 1 h and incubated for 24 h at 37 °C in the incubation chamber. Inhibition zones were measured using Vernier caliper and are expressed as the ‘mm’ zone of inhibition. Ciprofloxacin was used as a positive control (10 µg/mL). All the experiments were performed in triplicate.

### 4.10. MDA Contents

MDA (malondialdehyde) contents were calculated as described by Heath and Packer [63]. Plant samples (leaves and roots samples) of 500 mg were homogenized with 2 mL TCA (5%), and then we centrifuged the mixture at 10,000× *g* for 15 min. The supernatant in 1 mL volume was mixed with 1 mL TBA (0.5%) in 20% TCA, and the mixture was incubated at 95 °C for 30 min. Subsequently, the mixture was instantly cooled in an ice bath, centrifuged at 10,000× *g* for 5 min, and with the help of UV spectrophotometer absorbance was monitored at 532 and 600 nm. The non-specific value at 600 nm absorption was subtracted, and the total MDA contents were determined from its extinction coefficient at 155 mM^−1^cm^−1^.

### 4.11. Hydrogen Peroxide (H_2_O_2_)

H_2_O_2_ (hydrogen peroxide) levels were assessed according to Junglee et al. [64]. Fresh root and leaf samples of 500 mg were homogenized in 5 mL of 0.1% tri-chloroacetic acid (TCA) solution in an ice bath. Subsequently, the homogenate was centrifuged at 12,000× *g* for 15 min. A total of 1 mL of the supernatant, 0.5 mL of 10 mM K-phosphate buffer (pH¼7.0), and 1 mL of 1 mM potassium iodide (KI) were mixed in the test tube, and absorbance was taken at 390 nm by a UV spectrophotometer. Total H_2_O_2_ contents were calculated and expressed as µmol of H_2_O_2_ g ^−1^ fresh weight (FW).

### 4.12. Electrolyte Leakage

EL (electrolyte leakage) was determined according to the method of Lutts et al. [65]. Plant samples of leaves and roots were separately sliced into minute fractions equal to 5 mm and incubated for 24 h on rotary at 24 °C. Afterward, the preliminary EC1 was measured, and again the samples were kept in the oven for 120 min at 90 °C, collected, and cooled at 25 °C, and the second EC2 was calculated. Total EL value was measured with the help of the following equation:

EL (%) = (EC1/EC2) × 100(6)

### 4.13. Antioxidant Enzyme Extraction

Antioxidant enzymes of leaf and root samples were determined spectrophotometrically by using a pre-cooled mortar and pestle. Briefly, 500 mg fresh samples were homogenized in to 0.5 mL ice-cold K-phosphate buffer (100 mM and pH 7.3) containing EDTA (0.1 mM), phenylmethylsulfonyl fluoride (1 mM), and 3.65% polyvinylpyrrolidone. The homogenate was centrifuged for 15 min at 15,000× *g* and used for enzymatic analyses. The whole procedure was conducted at 4 °C.

### 4.14. Enzyme Determinations

SOD activity (EC 1.15.1.1) was measured according to Ries procedure with slight modification [46]. The reactant mixture contained 50 mM K-phosphate buffer (pH¼ 7.5), 35 µM nitro-blue tetrazolium (NBT), 10 mM methionine, 0.61 mM EDTA, 2.5 μM riboflavin, and 0.21% enzyme extract in a 3 mL final volume. The mixture was incubated at 28 °C under fluorescent light for 30 min. After incubation, absorbance was measured at 560 nm.

CAT activity (EC 1.11.1.6) was determined following the Aebi procedure [66] by observing the decrease in absorbance at 240 nm for 60 s. Briefly, the reactant mixture comprised 50 mM potassium phosphate buffer (pH 7.1), 15 mM H_2_O_2_, and 0.32% enzyme extract in a 3 mL final volume. The reaction was initiated by adding H_2_O_2_ to the reactant mixture, and CAT activity was calculated from the extinction coefficient, i.e., 39.4 mM^−1^cm^−1^.

GPX activity was calculated according to the procedure of Hossain et al. [67] by observing the decrease in absorbance at 340 nm for 60 s. The reaction mixture comprised 50 mM sodium phosphate buffer (pH¼ 7.2), 0.1 mM NADPH, 0.1 mM FeSO_4_, 0.1 mM H_2_O_2_, 0.1 mM EDTA, 0.1 mM (GSH), 0.1 unit of (GR), and 0.65% enzyme extract in a final volume of 3 mL. The reaction was initiated by adding enzyme extract, and GPX activity was measured using its extinction coefficient of 6.62 mM^−1^ cm^−1^.

POD activity was determined following the method of Wu et al. [68], using guaiacol as the substrate. The reaction mixture included 50 mM K-phosphate buffer (pH ¼ 7.3), 1% (w/v), 0.1 Mm H_2_O_2_, 0.1 Mm guaiacol, and 0.71% enzyme extract in a total volume of 3 mL. The reaction was initiated by adding guaiacol to the mixture, and absorbance was measured at 470 nm.

### 4.15. Elemental Analysis

Elemental analysis was done by following the procedure of Bankaji et al. [69]. The oven-dried samples (0.5 g) were ground into fine powder using a mortar and pestle and digested in acid mixture (HNO_3_ + HClO_4_) in a 5:1 proportion. After digestion, the samples were analyzed by ICP-OES (Optima-8300 DV; PerkinElmer, Inc., Waltham, MA, USA).

### 4.16. Statistical Analysis

The experiment was performed using a completely randomized design (CRMD). Data were recorded in the form of triplicates and analyzed by one-way analysis of variance (ANOVA) using statistical software package SPSS V. 21.0 (SPSS, Chicago, IL, USA). Mean separations were executed by Duncan’s multiple range tests. As compared to control, the percent inhibition/stimulatory effect were checked using the following formula), and significant differences were considered using different statistical letters/bars at *p* ≤ 0.05.

(7) $$\mathrm{Percentage}\;\left(\%\right)\;=\frac{\mathrm{Control}\;-\;\mathrm{Treatment}}{\mathrm{Control}}\times 100$$

## 5. Conclusions

This research project aimed to determine the effect of Cd and Cu on the growth, physio-chemicals, and medicinal changes in the medicinal plant pigeon pea. Cadmium (Cd)- and copper (Cu)-contaminated soil significantly influenced the morphological, biochemical, and physiological features of *C. cajan* L. Physiological activities such as chlorophyll a and b content, net photosynthesis, transpiration rate, and stomatal conductance were significantly declined in association with nutrient reduction among plants tissues with increasing Cd and Cu concentrations in the soil, leading to its growth inhibition. Pigeon pea experienced severe oxidative injuries under Cd and Cu stress, as measured in an elevated amount of MDA content, hydrogen peroxide, and electrolyte leakage. Cd and Cu at high concentrations suppressed phenolic and flavonoid biosynthesis that altered the medicinal efficiency of pigeon pea, as assessed in reduced antibacterial and antioxidant activates (DPPH and FRAP assays). Antioxidant enzymes—SOD, POD, CAT, and GPX—along with proline contents were significantly enhanced with increasing Cd and Cu concentration to minimize the oxidative damage caused. On the basis of results obtained in our present study, it could be concluded that the cultivation of pigeon pea in Cd and Cu amended soil could inhibit plant growth and alter its medicinal properties.

## Figures and Tables

**Table 1:** The combine effects of cadmium (Cd) and copper (Cu) on the roots’ and shoots’ fresh and dry weights (g) and root and shoot length (L) in the pigeon pea plants. Various statistical letters represent significant differences from control.

Treatment	Roots			Shoots		
	Fresh Weight (g)	Dry Weight (g)	Length (cm)	Fresh Weight (g)	Dry Weight (g)	Length (cm)
Control	5.55 ± 1.0 ^d^	2.61 ± 0.23 ^d^	23.40 ± 1.1 ^d^	9.45 ± 0.55 ^b^	5.11 ± 0.43 ^d^	91.40 ± 1.15 ^d^
Cd 20 + Cu 30 mg/kg	5.10 ± 0.99 ^c^	2.42 ± 0.60 ^c^	21.40 ± 0.85 ^c^	8.75 ± 0.77 ^b^	4.91 ± 0.89 ^c^	86.10 ± 0.85 ^c^
Cd 40 + Cu 60 mg/kg	4.72 ± 0.7 ^b^	2.11 ± 0.32 ^b^	18.45 ± 0.77 ^b^	7.11 ± 0.01 ^a^	4.01 ± 1.02 ^b^	80.50 ± 0.5 ^b^
Cd 60 + Cu 90 mg/kg	3.92 ± 1.23 ^a^	1.82 ± 0.94 ^a^	13.45 ± 0.9 ^a^	6.21 ± 0.5 ^a^	3.21 ± 0.93 ^a^	73.40 ± 0.23 ^a^

**Table 2:** Total flavonoid, phenolic, and proline contents of pigeon pea under different cadmium and copper concentrations. Various statistical letters represent significant differences from control.

Treatment	Flavonoids (mg/g DM GAE)	Phenols (mg/g DM Rutin)	Proline (µg/g FW)
Control	32.35 ± 0.34 ^c^	55.51 ± 0.91 ^c^	20.43 ± 0.15 ^a^
Cd 20 + Cu 30 mg/kg	42.40 ± 0.12 ^d^	68.53 ± 0.21 ^d^	45.41 ± 0.18 ^b^
Cd 40 + Cu 60 mg/kg	26.45 ± 0.39 ^b^	45.42 ± 0.45 ^b^	70.40 ± 0.71 ^c^
Cd 60 + Cu 90 mg/kg	17.41 ± 0.78 ^a^	32.46 ± 0.81 ^a^	90.47 ± 0.12 ^d^

**Table 3:** The anti-bacterial activities of pigeon pea leave crude extracts of different Cd and Cu treatments through the disc diffusion approach. Various statistical letters represent significant differences from control.

Antibacterial Activities	Inhibition Zone (mm)		
	Gram-Positive Bacteria	Gram-Negative Bacteria	
	*S. aureus*	*E. coli*	*S. thyphi*
Control	11.60 ± 0.86 ^c^	10.52 ± 0.57 ^c^	12.36 ± 0.97 ^c^
Cd 20 + Cu 30mg/kg	12.93 ± 0.34 ^d^	11.22 ± 0.77 ^d^	13.75 ± 0.58 ^d^
Cd 40 + Cu 60 mg/kg	10.10 ± 0.57 ^b^	9.92 ± 0.33 ^b^	10.91 ± 0.11 ^b^
Cd 60 + Cu 90mg/kg	9.65 ± 0.83 ^a^	8.44 ± 0.45 ^a^	8.21 ± 0.63 ^a^
Ciprofloxacin	17.61 ± 0.88 ^e^	19.59 ± 0.71 ^e^	21.29 ± 0.77 ^e^

## Data Availability

All datasets on which the conclusions of the manuscript rely are presented in the paper.

## List of Abbreviations and Acronyms

Cadmium (Cd), Copper (Cu), Superoxide dismutase (SOD), Catalase (CAT), Peroxidase dismutase (POD), Glutathione peroxidase (GPX), 1,1-diphenyl-2-picrylhydrazyl (DPPH), ferric-reducing antioxidant potential (FRAP), Malondialdehyde contents (MDA), lectrolyte leakage (EL), Hydrogen peroxide (H_2_O_2_), Reactive oxygen species (ROS), Photosynthetic photon flux density (PPFD), *Staphylococcus aureus* (*S. aureus*), *Escherichia coli* (*E. coli*), *Staphylococcus typhi* (*S. thyphi*), Sodium hypochlorite (NaOCl), Net photosynthesis (Pn), Absorbance (Abs), Sodium hydroxide (NaOH), Sodium nitrate (NaNO3), Aluminum chloride (AlCl3), 2,4,6-Tripyridyl-S-triazine (TPTZ), Hydrocloric acid (HCL), Ferric chloride (FeCl3), Trichloroacetic acid (TCA), Ethylenediamine tetraacetic acid (EDTA), Nitro-blue tetrazolium (NBT), Nicotinamide adenine dinucleotide phosphate (NADPH), Glutathione (GSH), Inductively coupled plasma–optical emission spectrometry (ICP-OES), Millimolar (mM), Microgram (μg).
